# Unleashing the potential of artificial intelligence in infectious diseases

**DOI:** 10.1093/nsr/nwaf004

**Published:** 2025-01-08

**Authors:** Hang-Yu Zhou, Yaling Li, Jiaying Li, Jing Meng, Aiping Wu

**Affiliations:** State Key Laboratory of Common Mechanism Research for Major Diseases, Suzhou Institute of Systems Medicine, Chinese Academy of Medical Sciences & Peking Union Medical College, China; Key Laboratory of Pathogen Infection Prevention and Control (Peking Union Medical College), Ministry of Education, China; Development Strategy and Cooperation Center, Zhejiang Lab, China; Zhejiang Laboratory of Philosophy and Social Sciences - Laboratory of Intelligent Society and Governance, Zhejiang Lab, China; State Key Laboratory of Common Mechanism Research for Major Diseases, Suzhou Institute of Systems Medicine, Chinese Academy of Medical Sciences & Peking Union Medical College, China; Key Laboratory of Pathogen Infection Prevention and Control (Peking Union Medical College), Ministry of Education, China; State Key Laboratory of Common Mechanism Research for Major Diseases, Suzhou Institute of Systems Medicine, Chinese Academy of Medical Sciences & Peking Union Medical College, China; Key Laboratory of Pathogen Infection Prevention and Control (Peking Union Medical College), Ministry of Education, China; State Key Laboratory of Common Mechanism Research for Major Diseases, Suzhou Institute of Systems Medicine, Chinese Academy of Medical Sciences & Peking Union Medical College, China; Key Laboratory of Pathogen Infection Prevention and Control (Peking Union Medical College), Ministry of Education, China

Artificial intelligence (AI) has emerged as a transformative force across various scientific disciplines. In life sciences, AI has facilitated advancements in genomics, protein structure prediction, protein design and drug discovery [1].

In the realm of infectious diseases, the rapid expansion of AI applications is revolutionizing pandemic prediction, pathogen detection and the design of treatment strategies [2]. For instance, AI-driven early-warning systems like HealthMap utilize natural language processing to analyse real-time data from various web sources (Fig. 1A), enabling the swift identification of discussions of emerging pathogens [3]. Furthermore, AI-based systems can differentiate between similar pathogens, such as SARS-CoV-2 and influenza viruses (Fig. 1B), enabling more targeted public health responses [2]. Beyond forecasting, AI-driven models are instrumental in therapeutic development (Fig. 1C), efficiently screening vast chemical libraries to identify potential drug candidates and predicting drug–target interactions with high precision [4]. Compared with traditional bioinformatics and modeling approaches, AI algorithms excel in managing larger data sets, uncovering intricate patterns and delivering more accurate predictive outcomes.

**Figure 1.**
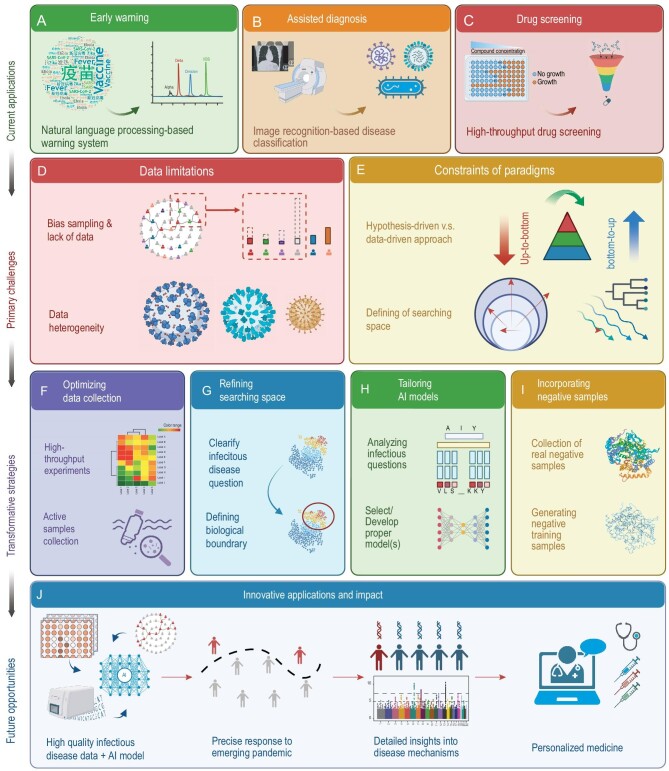
Advancing infectious disease research through AI: from challenges to future frontiers. (A) Employing natural language processing for early detection of infectious disease outbreaks. (B) Utilizing image recognition algorithms for disease classification. (C) Facilitating high-throughput drug screening with AI-based methods. (D) Addressing challenges arising from data limitations. (E) Navigating paradigm shifts in research approaches. (F) Mitigating data collection bias through optimized data acquisition strategies. (G) Refining problem spaces to align with specific biological contexts rather than broad mathematical domains. (H) Selecting AI models based on targeted biological questions instead of defaulting to the latest or largest available options. (I) Enhancing AI model performance and accuracy in infectious disease studies through the deliberate inclusion of negative samples. (J) Revolutionizing disease modeling, surveillance and personalized treatment strategies by integrating high-throughput technologies, single-cell genomics, large language models and precision medicine approaches. The original draft was created in BioRender.

This perspective aims to elucidate the potential of AI in transforming infectious disease research and application. We outline current challenges and propose strategic approaches to overcome these barriers, highlighting the importance of clearly defining specific scientific questions and aligning AI applications with the unique characteristics of infectious diseases.

## CURRENT CHALLENGES: DATA LIMITATIONS AND RESEARCH PARADIGMS

### Data limitations

Sampling bias represents a fundamental limitation in pathogen surveillance and study (Fig. 1D), manifesting through four critical aspects: natural selection bias, sample size bias, geographic distribution bias and data accessibility bias. In viral evolution studies, natural selection bias emerges as existing data sets predominantly reflect successful variants while lacking negative samples, thus hindering the development of comprehensive models that capture the full spectrum of viral diversity and adaptability. The sample size bias is equally problematic—while SARS-CoV-2 has generated abundant data, most other pathogens suffer from insufficient sample sizes, which significantly impedes the training of large-scale AI algorithms. Furthermore, data from low- and middle-income countries frequently remain under-digitized or inaccessible [5], creating substantial obstacles for developing representative AI models. These geographical disparities in data availability and quality ultimately compromise our ability to build truly comprehensive analytical frameworks.

Beyond sampling biases, the intricate complexity of pathogen diversity poses additional challenges. Infectious diseases encompass a wide array of pathogens—including viruses, bacteria, fungi and parasites—each with unique biological characteristics. This complexity is particularly evident in cases of insufficient genomic data, where modeling accuracy becomes a significant concern. For instance, vector-borne diseases such as dengue fever require complex transmission models that must integrate various factors including environmental data, vector behavior and human activity patterns [6], all of which are difficult to quantify and incorporate into AI models.

Moreover, the inherent heterogeneity of pathogens and their dynamic interactions with hosts and environments adds another layer of complexity. Pathogen transmission, mutation and evolution processes are partly stochastic and inherently dynamic, necessitating sophisticated models that are capable of adapting to both changing conditions and sparse data availability.

### Obstacle in research paradigms

Current research paradigms in infectious diseases present additional challenges for AI integration (Fig. 1E). The traditional hypothesis-driven approach, while valuable, may not fully leverage the potential of AI for data-driven discovery. There is often a disconnect between the vast mathematical space that is explored by AI algorithms and the more constrained biological space that is relevant to infectious diseases. This mismatch can lead to inefficient exploration of searching space, a waste of computational resources and potentially irrelevant or misleading results.

Moreover, the current paradigm often focuses on specific pathogens or disease outcomes, potentially overlooking broader patterns or interdependencies in infectious disease dynamics. AI models that are trained on such narrowly defined data sets may struggle to generalize across different pathogens or disease scenarios.

This limitation is particularly problematic given the potential for emerging infectious diseases and the need for flexible, adaptable modeling approaches.

Another paradigmatic challenge lies in the integration of multiscale data, from molecular omics to population-level epidemiology. Current research often compartmentalizes these scales, but AI offers the potential to bridge these divides and create more holistic models of infectious disease dynamics. However, the realization of this potential requires rethinking how we structure and integrate data across different biological and epidemiological scales.

## TRANSFORMATIVE STRATEGIES: HARNESSING THE POTENTIAL OF AI

### Optimizing data collection and integration

Biological data are often small in scale, imbalanced and influenced by the subjective willingness of sampled individuals. To address these issues, additional infectious disease-associated surveillance and detection data need to be integrated (Fig. 1F). For instance, emerging methods such as wastewater surveillance and digital tracing offer more comprehensive and unbiased data, enhancing the robustness of AI models [7]. High-throughput experimental techniques such as deep mutational scanning (DMS) systematically assess the functional impact of protein mutations in the lab, enriching data sets for AI model training and mitigating data insufficiency [8].

### Redefining the searching space

In studying infectious diseases, the problem space is considerably narrower than the theoretical mathematical space that is often targeted by traditional AI methods (Fig. 1G). Precisely defining biological questions and the feature space are crucial. Instead of pursuing broad, all-encompassing scopes, we should focus on setting reasonable analytical boundaries based on practical research needs. For example, while k-mer analysis attempts to capture as much potential range as possible, its efficacy may be limited when dealing with constrained biological data sets. Compressed encoding methods such as byte pair encoding have re-emerged, saving computational resources and aligning the target space with the specific biological problem [9]. This shift underscores the importance of selecting encoding strategies that are consistent with the biological context.

Another example is from the prediction of viral evolution. Rather than concentrating solely on predicting specific strains, efforts should aim to characterize the possible mutational space of viruses and identify constraints on their evolutionary trajectories [10]. This is because viruses evolve within a limited space that is shaped by factors such as pathogen properties, host interactions and environmental pressures. Leveraging AI to identify these spatial constraints could be key to predicting future viral mutations. Integrating evolutionary principles and domain knowledge into AI model design can lead to models that are both biologically plausible and interpretable.

### Tailoring AI models to biological questions

The selection of AI models should be driven by specific biological questions rather than simply adopting the latest or largest available models (Fig. 1H). While transformer-based methods have shown success in various fields, simpler approaches often prove equally effective for specific tasks. For example, Zhang *et al.* demonstrated the effectiveness of a grid search in mRNA design, successfully optimizing both protein expression and thermostability [11]. This highlights the importance of matching modeling approaches to specific biological objectives, particularly in complex scenarios such as vaccine design, in which multiple goals must be balanced.

### Incorporating negative samples

A significant limitation in infectious disease research is the scarcity of negative samples (Fig. 1I). Recent research by Hou *et al.* has highlighted how the incorporation of negative samples—particularly those that share similarities with the target proteins—can improve AI models for metagenomic viral identification [12]. This emphasizes the importance of deliberately collecting or generating negative samples and considering their potential impact on model performance.

## FUTURE OPPORTUNITIES: INNOVATIVE APPLICATIONS AND IMPACT

Looking ahead, the next breakthroughs at the intersection of AI and infectious disease research may emerge in fields involving high-throughput technologies, propelled by the rapid advancement of experimental techniques and decreasing computational costs (Fig. 1J). Technologies such as DMS provide valuable data by comprehensively evaluating the functional impact of protein mutations, supporting AI model development. In infectious disease surveillance, advancements in wastewater monitoring and digital tracing enhance early detection and response capabilities.

With the progression of single-cell technologies, disease modeling and digital twins are poised to rise swiftly. Single-cell multi-omics offers detailed insights into cellular heterogeneity and disease mechanisms [13]. The integration of AI—particularly generative models such as GPT—can augment our capacity to model complex biological systems and predict disease progression.

The rapid development of large language models (LLMs) presents new avenues. Methods such as TxGNN [14] and scGPT [15] have achieved zero-shot downstream tasks through interaction with LLMs, showcasing immense potential in this burgeoning field. These foundational models can adapt to various tasks in infectious diseases without extensive retraining, which enhances efficiency and applicability.

As AI continues to evolve, its integration with infectious disease research holds unprecedented opportunities in personalized medicine. The ability of AI to analyse data at the individual level can facilitate tailored diagnostics and therapeutics. Advancements in AI computational power and data availability have significantly reduced the cost of individual-level analyses. Personalized precision medicine in infectious diseases—which encompasses data collection, diagnostics and individualized treatment—may become the next frontier for breakthroughs.

The integration of AI with infectious disease research promises transformative changes in pandemic preparedness, response and management. By enhancing data collection methods, adopting innovative AI approaches, integrating domain knowledge and addressing interpretability and ethical considerations, we may overcome current challenges in applying AI to infectious diseases. The future holds vast opportunities in personalized precision medicine, advanced disease modeling and prediction, drug and vaccine development and real-time infectious disease monitoring.

Continued advancements in high-throughput experimental techniques and reductions in computational costs will deepen the integration of AI and infectious diseases, leading to breakthroughs in understanding pathogen evolution, host–pathogen interactions and disease dynamics. Interdisciplinary and cross-sector collaborations are crucial to fully realizing the transformative potential of AI in global health security. The complementary integration of AI with traditional methods will better equip humanity to tackle future infectious disease threats, safeguarding health and well-being globally.
